# New beam-based models for fire-induced buckling analysis of class 4 steel columns

**DOI:** 10.1016/j.heliyon.2024.e26951

**Published:** 2024-03-02

**Authors:** Myriam R. Pallares-Muñoz, Ignacio Paya-Zaforteza, Antonio Hospitaler-Perez

**Affiliations:** a Universidad Surcolombiana, Cra 1 Aven. 26, 410010, Neiva, Colombia; b Universitat Politècnica de València, ICITECH, Camino de Vera S/N, 46022, València, Spain

**Keywords:** Instability, Fibre-type numerical models, Flexural-torsional buckling, Thin-walled steel members, Class 4 steel cross-section, Residual stresses, Geometric imperfections, Nonlinear mechanical analysis with imperfections, Steel members under fire

## Abstract

Steel cross-sections with thin walls are vulnerable to fire-induced buckling instability, which reduces their load-bearing capacity. Eurocode 3 design provisions have been found inadequate, leading to alternative methods such as effective design strategies and advanced structural models built mostly with shell FE, which can be complex. For Class 4 steel beam-columns subjected to fire conditions, beam-type modelling to predict the Flexural-Torsional Buckling (FTB) strength has been proposed as an alternative approach, but it has not yielded satisfactory results for large compressive load eccentricities. This paper presents two new low computational cost modelling strategies based on Timoshenko's beam FE to address this issue: the Single beam-column Model (SbcM) and the Cruciform beam-column Model (CbcM). The first consists of a single line of beam FE, while the second uses a grid of beam FE for more flexibility. Both strategies effectively simulate the FTB behaviour in Class 4 steel beam-column during a fire, offering quicker computations compared to shell models. Still, the single-line model is favoured for its simplicity, making it more efficient in analysing complex fire engineering problems.

## 1 Introduction

Recent advances in steel fabrication technologies have led to the construction of structures with significant weight reductions thanks to the combined use of high-strength steel and thin-walled cross-sections. This practice makes use of slender steel cross-sections, which are prone to local buckling failures, especially under fire, as demonstrated by the fire-provoked failures observed in tall steel-framed buildings such as Wilton Paes de Almeida (Brazil, 2018) [1], Windsor Tower (Madrid, 2005) [2], Beijing Television Cultural Centre (Beijing, 2009) [3] and World Trade Centre 1,2,7 (New York, 2001) [4,5]. Buckling was also the predominant failure mode in other less recent high-rise steel-framed fires, for example, the fire at One Meridian Plaza (Philadelphia, 1991), the First Interstate Bank (Los Angeles, 1988), and the Winecoff Hotel (Atlanta, 1946). In all these steel-framed buildings, exposure of the thin-walled elements to fire resulted in the complete structural collapse of the building or a significant portion thereof. Thin-walled structural elements are also employed in other steel structures, such as long-span bridges, industrial facilities, and hangars. Hence, an in-depth study of the buckling phenomenon in these cross-sections is essential to ensure their correct performance in fire conditions. Thin-walled structural steel elements are typically fabricated from slender plates, with depths spanning from 25 to 305 mm and a maximum thickness of 13 mm [6]. In the event of a fire, these elements commonly experience failure due to a combination of global and local buckling. For example, when thin-walled beams are exposed to fire, failure is induced by Lateral-Torsional Buckling (LTB), while thin-walled slender steel columns fail due to Flexural-Torsional Buckling (FTB). In both cases, local buckling can also appear in the thin steel plates of the web or flanges subjected to high compressions. Hence, this occurrence must be considered an integral aspect of the design of steel structural components. The current Eurocode 3 [7] design method accounts for local buckling in determining the ultimate strength of thin steel plates at high temperatures. That is achieved by evaluating the effective width of the compressed sections exposed to fire. However, some authors [8,9] consider the Eurocode 3 approach unsafe, and many studies have paid attention to the study of the problem. These studies, summarised in Ref. [8], have included analytical design expressions and experimental and numerical simulations with equivalent design formulations. However, numerical simulations have predominantly employed shell and solid elements [[10], [11], [12], [13], [14], [15], [16], [17], [18], [19], [20], [21], [22], [23], [24]] due to their inherent ability to replicate the buckling phenomena (global and local) in structural steel elements featuring thin-walled cross-sections. The challenge with shell FE is that their modelling and CPU times are often considerable, making them computationally expensive for more advanced and complex fire engineering studies involving large numbers of simulations. Therefore, it is essential to create simplified beam-based models that are as reliable as shell-based models, making these highly nonlinear analyses computationally cost-effective. Notwithstanding this requirement, beam FE have been used less frequently in instability simulations of fire-exposed steel members [[25], [26], [27], [28], [29], [30], [31]], with most of the studies focusing on columns [[28], [29], [30]] and fewer on beams [27,31].

Furthermore, the formulations proposed to account for local buckling are based on effective methods reducing the load-carrying capacity of the thin-walled steel member under fire exposure. For example, the eﬀective cross-section methods [9,13,[32], [33], [34], [35], [36]] decrease the steel cross-section area, and the strain-based calculation methods modify the temperature-dependent constitutive model of steel [35]. The stress-based method is another effective strategy to determine the resistance to fire of such specific cross-sections. An example is using an equivalent stress-based model within Bernoulli beam FE, which was introduced in Ref. [25] and subsequently enhanced in Ref. [30]. This model implemented in Safir [37] showed computational time savings compared to traditional shell models, and its predictions were satisfactory for small compressive load eccentricities but conservative for large ones. Thus, the model is still under development [30]. Other recent approaches with beam-type elements include a new Timoshenko element to account for torsional buckling effects in steel elements subjected to fire [38], as well as two novel proposals for predicting the LTB strength in steel beams featuring Class 4 cross-sections under fire exposure [27,31]. These proposals based on Timoshenko beam FE and nonlinear analysis with imperfections were validated with tests on FIDESC4 Class 4 steel beams with constant cross-section [10,21]. However, one of the models overestimated ultimate deflection and cumulative strain energy, failed to reproduce local buckling accurately, and produced high computational costs. For this reason, such a strategy is still being improved.

In contrast, fire instability in steel columns with thin-walled sections and sizeable eccentricities of the compressive load is a highly nonlinear problem of considerable complexity where beam-based models have not given predictions close to the actual behaviour. However, the low computational cost of beam-based models is crucial for decreasing the computational time of more complex fire design problems, which is required to foster new progress in topics such as structural optimisation or the probabilistic analysis of structures under fire. With this in mind, two new strategies for modelling fire-induced failure in thin-walled steel beam-columns are proposed. These strategies accurately predict the FTB strength of these members with significant reductions in CPU time for both small and large compressive load eccentricities, allowing faster and more accurate analysis of complex fire engineering problems.

The two new strategies, denominated Single beam-column Model (SbcM) and Cruciform beam-column Model (CbcM), are fibre-type models discretised with Timoshenko beam FE with seven DOF. SbcM is a row of I-beam FE placed in the load axis of the column. At the same time, CbcM idealises the steel beam-column as a gridded arrangement of rectangular-beam FE, allowing more flexibility and capturing local instability in highly compressed regions with more detail than SbcM. These numerical strategies use the steel member's nominal flexural and axial stiffness (not their reduced values) and consider the induced thermal compression stresses due to temperature increments. Unlike the effective approaches, SbcM and CbcM do not reduce the cross-section area or modify the Eurocode 3 constitutive material law at elevated temperatures. Instead, they use discretisation models with boundary conditions adjusted to the phenomenon's reality in thin-walled, slender steel columns. Likewise, they include geometrical and material non-linearity, suitable initial geometric and material imperfections, load-induced strains and the action of gravity. These models also incorporate the nonlinear buckling analysis and the post-buckling solution. Furthermore, they are validated with the numerical and laboratory results of fire tests 3 and 6 conducted on Class 4 constant cross-section columns of the FIDESC4 investigation [10] and with the numerical results of FIDESC4 fire test 3 presented in Ref. [30].

The modelling approaches of SbcM and CbcM, designed to predict the FTB in slender Class 4 steel section columns under fire, differ from the methodologies detailed in Refs. [27,31] for assessing the LTB in Class 4 steel section beams under fire because both phenomena have inherent physical and conceptual variations. As a result, SbcM and CbcM techniques are tailored to the attributes of FTB, integrating crucial factors such as support conditions, including the axial restraint (confinement), thermal and mechanical loading conditions, and the initial states typical of slender Class 4 steel section columns exposed to fire.

This document is organised into seven sections. Section 2 provides information on fire tests 3 and 6, which were conducted on Class 4 constant cross-section columns of the FIDESC4 investigation and used for validation purposes. Section 3 describes the SbcM and CbcM modelling strategies, while Section 4 provides a detailed methodology for applying the complete GMNIA analysis in SbcM and CbcM. Section 5 explains how the proposed modelling strategies were implemented. In Section 6, the validation results of the proposed strategies compared to both laboratory and numerical data are presented. Lastly, Section 7 concludes the numerical investigation, outlines its limitations and discusses possible areas for future research.

## 2 Description of the fire tests for validating the strategies

The SbcM and CbcM modelling strategies were validated using the experimental and numerical results of tests 3 and 6 on Class 4 constant cross-section columns of the FIDESC4 investigation [10]. Therefore, this section discusses the primary features of the fire experiments.

**Fig. 1:**
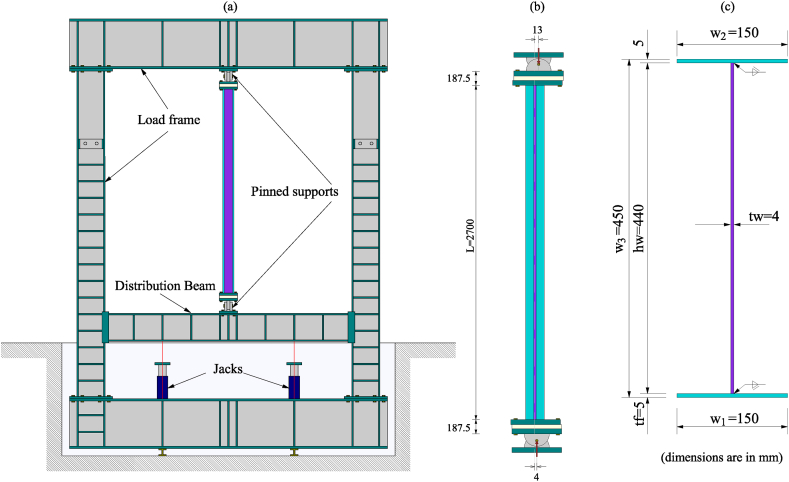
Test 3 [10]: (a) Setup, frontal view. (b) Eccentricities, lateral view. (c) I-welded cross-section.

**Fig. 2:**
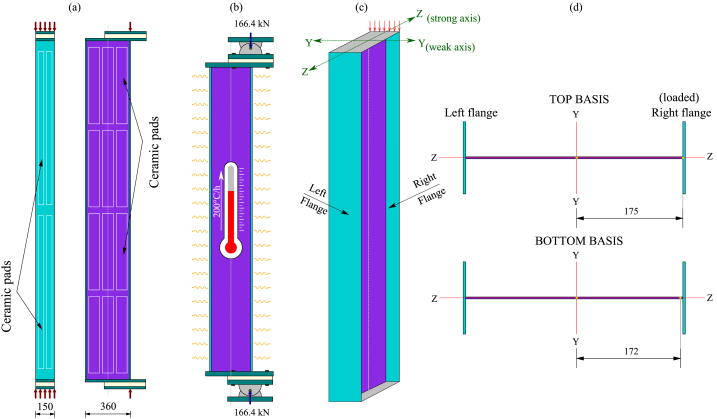
Test 3 [10]: (a) Location of resistors. (b) Mechanical and thermal loading. (c) Load axes. (d) Eccentricities.

**Fig. 3:**
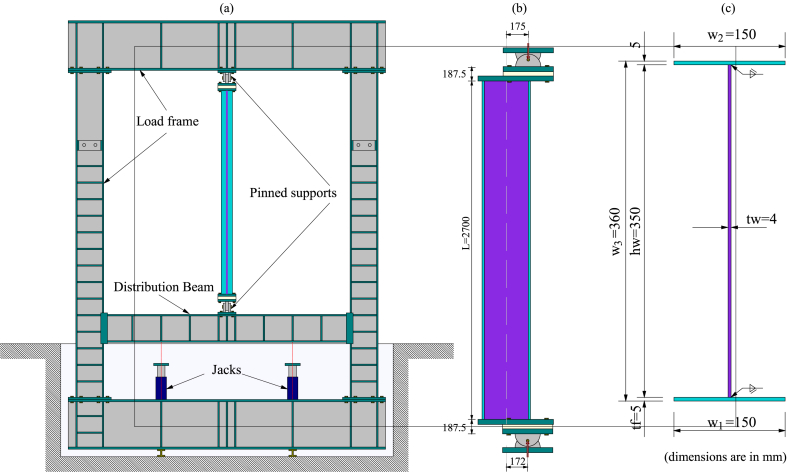
Test 6 [10]: (a) Setup, frontal view. (b) Eccentricities, lateral view. (c) I-welded cross-section.

**Fig. 4:**
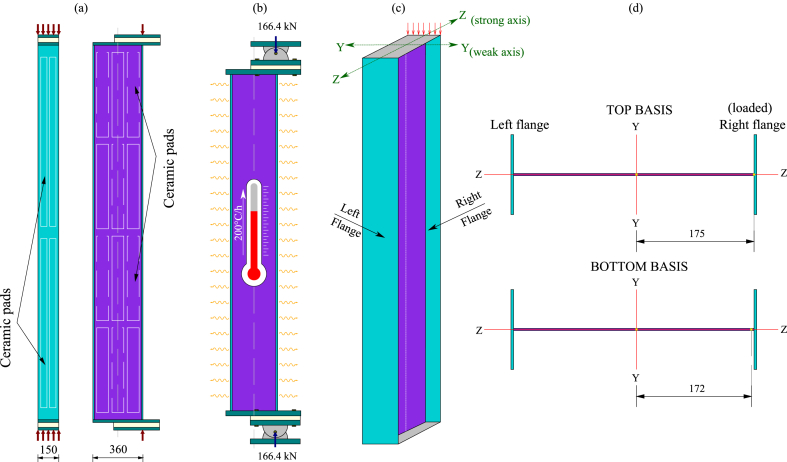
Test 6 [10]: (a) Location of resistors. (b) Mechanical and thermal loading. (c) Load axes. (d) Eccentricities.

The experiments were conducted on pinned-supported, slender, built-up welded columns simulating beam-columns featuring Class 4 cross-sections. The elements were loaded at room temperature until reaching the test load and then heated, maintaining the load until mechanical failure. The test load at room temperature was defined as the equivalent of the failure load at 450 °C calculated according to Eurocode 3 [7]. In this way, the tested columns were first loaded axially and then progressively heated, providing a uniform increase in the temperature at a constant heating rate of 200 °C per hour along the column (L = 2700 mm) until failure. The tests were designed without lateral restraint located along the weak axis, looking for a failure induced by a global buckling along with local buckling of flange walls. The setup, geometrical definition, and cross-sections of both tested columns are presented in Fig. 1, Fig. 2, Fig. 3, Fig. 4 (Fig. 1, Fig. 2 correspond to test 3, while Fig. 3, Fig. 4 correspond to test 6). Fig. 1, Fig. 3a show the test setup in a front view, Fig. 1, Fig. 3b depict the compressive load eccentricities in a side view, and Fig. 1, Fig. 3c illustrate the welded I-section. Meanwhile, Fig. 2, Fig. 4a represent the location of resistors, Fig. 2, Fig. 4b illustrate the mechanical and thermal loading, Fig. 2, Fig. 4c show the load axes and Fig. 2, Fig. 4d show the eccentricities.

Test 3 was subjected to compressive axial load with a small eccentricity. Test 6 was subjected to combined axial compression and bending caused by a large eccentricity. The eccentricities between the load and the supports were intended to create an even distribution of bending moments along the columns. In test 3, a maximum eccentricity of 5.0 mm about the weak axis was used to induce the failure mode while producing a small uniform bending moment distribution. However, the load was applied with an effective eccentricity at the bottom basis of 13.0 mm and the top basis of 4.0 mm, as shown in Fig. 1b. In turn, test 6 was set for an eccentricity of 177.5 mm about the strong axis. However, the load was applied with an effective eccentricity at the bottom basis of 172.0 mm and the top of 175.0 mm, as shown in Fig. 3b. These imprecisions resulted from slacks between the holes of the endplates, the holes of the pinned supports, and the thread bars used to fix the endplates to the pinned supports with different diameters.

The tests were mounted on a steel frame equipped with jacks that applied the necessary mechanical load by pushing up a lower distribution beam (see Fig. 1, Fig. 3a). The total length of the tests to be considered between the load application points includes the 2700 mm length of the column, the thickness of the two endplates (2×(40/2)), the two half thicknesses of the pinned supports (2×(265/2) mm) and the thickness of the insulating layers (2 × 35 mm). Therefore, the overlength at each extremity of the column is 187.5 mm, as shown in Fig. 1, Fig. 3b. Heating power units and several ceramic elements made it possible to heat the entire column. At the same time, the temperature was measured with thermocouples. Fig. 2, Fig. 4 illustrate the thermal and mechanical loads, the two load axes and the associated eccentricities.

The material properties of the plates used for flanges and the web were measured at ambient temperature. The average yield strength and elastic modulus values of the samples used in both tests and overlength material are listed in Table 1 [10]. The slendernesses (*λ*) in the strong and weak axes shown in Table 2 were established according to Eurocode 3 [7].

**Table 1:** Material properties.

Part of the column	Average Yield Stress (MPa)	Elastic Modulus (MPa)
Web	464.5	210,000
Flanges	404	210,000
Overlength	355	210,000

**Table 2:** Slendernesses of the plates.

*λ*	Test 3	Test 6
Strong axis	0.164	0.212
Weak axis	0.995	0.991

**Fig. 5:**
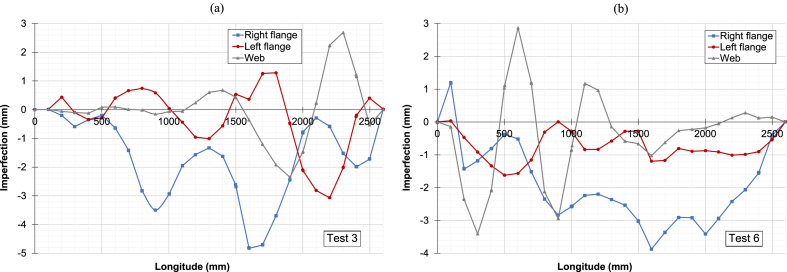
Local imperfection profiles: (a) Test 3. (b) Test 6.

Fig. 5 shows the plotting of local imperfections. Manual measurements using an aluminium rule were used to create the geometry of the entire column before each fire test. This process helped determine the imperfection profile along the web and flanges for tests 3 and 6, as shown in Fig. 5a and b, respectively [10]. The amplitudes, which are the maximum values of these profiles, were recorded for each test and can be found in Table 3 [10].

**Table 3:** Amplitude of imperfections.

Amplitude [mm]	Test 3	Test 6
Local - web	2.7	3.4
Local - left flange	3.0	1.6
Local - right flange	4.7	3.9
Global - weak axis	5.4	1.0

**Fig. 6:**
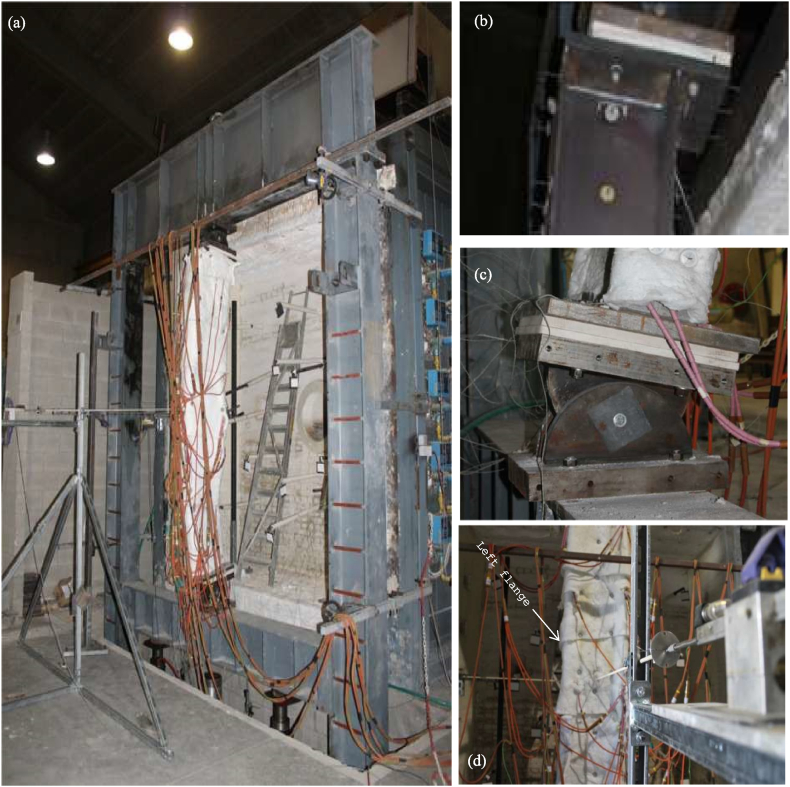
(a) Test setup in the laboratory and details. (b,c) Pinned support, endplates, and insulating material. (d) Transverse displacement measuring transducers [10].

Fig. 6 shows how a test is mounted in the laboratory. The experimental setup is illustrated in Fig. 6a. The column ends were fixed with pinned supports, allowing for rotation in only one direction. The vertical displacement was restrained at the bottom and released at the top, as shown in Fig. 6b and c. In addition, a thermal layer of the special insulating material was placed between the steel endplate of the tested column and the steel pinned support to ensure enough compression strength in the heated state (refer to Fig. 6b and c). Thus, the supports could not exceed a temperature of 200 °C. However, the tested column with endplates could be heated up to 600 °C. The mean temperature value was recorded throughout the experiment. The heating and loading process was stopped at 131 min in test 3 and 154 min in test 6. In the same order, the experimental failure temperatures were 452 °C and 530 °C. The global vertical shortening of the loaded column subjected to elevated temperatures was measured using transducers located in the lower zone of the load distribution beam, as shown in Fig. 6a. The displacement about the weak axis was obtained at half the web width. The strong axis displacement was measured at the centre of the left flange's width, as shown in Fig. 6d.

## 3 Modelling strategies and the finite element used

Most numerical simulations of thin-walled steel members, including those of the FIDESC4 research project [10], use shell FE that naturally capture local buckling but require high modelling and CPU times. This fact limits their use in fire engineering studies, especially in those related to structural optimisation and probabilistic modelling.

**Fig. 7:**
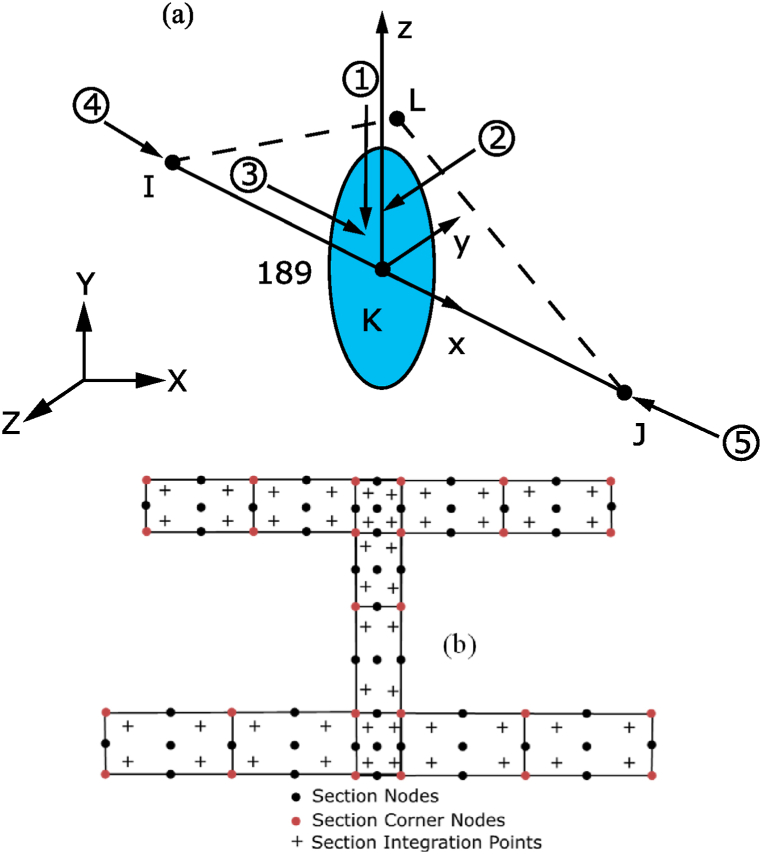
BEAM189 element: (a) Description. (b) Cross-section design [40].

Beam models are an alternative whose challenge is creating a good beam discretisation model with appropriate boundary conditions incorporating adequate geometrical and material imperfections. The paper introduces two modelling strategies, SbcM and CbcM, for analysing the behaviour of Class 4 beam-columns under elevated temperatures when subjected to FTB. These strategies are based on quadratic Timoshenko beam FE with seven DOF (see Fig. 7). Each element section comprises cells with nine nodes and four integration points, as shown (see Fig. 7a and b), which enhance the accuracy and modelling of nonlinear stress-strain relationships [26,39]. This element accommodates uniform temperatures and thermal gradients [40].

**Fig. 8:**
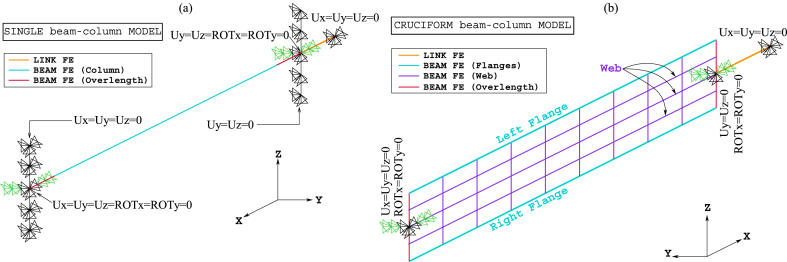
Idealisation of test 3: (a) SbcM. (b) CbcM.

**Fig. 9:**
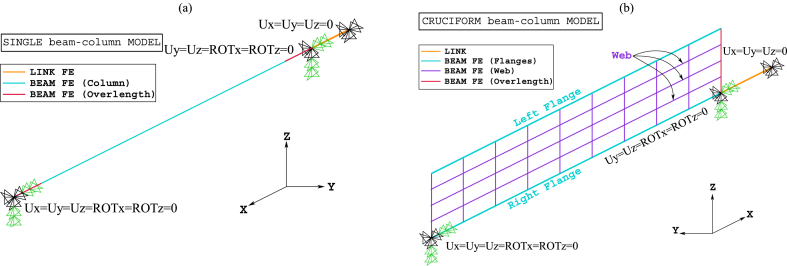
Idealisation of test 6: (a) SbcM. (b) CbcM.

Fig. 8, Fig. 9 show the idealisations of the SbcM and CbcM strategies for tests 3 and 6, respectively. The SbcM strategy discretises the column as a line of connected I-beam FE (see Fig. 8, Fig. 9a representing the SbcM for tests 3 and 6). In the CbcM, the column is defined as a cruciform arrangement of connected rectangular-beam FE (see Fig. 8, Fig. 9b representing the CbcM for tests 3 and 6) to better emulate the structural member response.

The distance between the two load application points in the models comprises the 2700 mm column length and an overlength that includes the thicknesses of the two steel endplates, the pinned supports, and the insulating material (see Fig. 1, Fig. 3b). This overlength is modelled as an additional vertical line of beam FE along the column axis, as shown brown-coloured in Fig. 8, Fig. 9. Moreover, a nonlinear compression link FE is included in both modelling strategies to represent the testing machine confinement created by the thermal expansion (see Fig. 8, Fig. 9). The link FE is positioned next to the column overlength on the load axis, and its length is arbitrary. For both proposed modelling strategies, a length of 200 mm was assumed, which is reasonable for large strains. Moreover, the cross-section area of the link FE is calculated with the proposed Equation (1) and easily calibrated for each model since it only slightly affects the deflection results near failure.

(1) $$A_{\text{link}}=\text{factor}\frac{F_{applied}}{F_{y}^{Steel}}$$

## 4 Methodology for FTB analysis of tested columns

FTB phenomenon occurring in thin-walled steel beam-columns exposed to fire is enveloped with complexities, including a correct definition of the boundary conditions, geometrical and material non-linearities, initial geometrical and material imperfections, thermal expansion effects, confinement-induced stresses, and the combination of load and temperature stresses. All these events imply that the problem must be solved by a static or dynamic geometric and materially nonlinear analysis, including imperfections (GMNIA). For this reason, the proposed modelling strategies presented in this paper use a static GMNIA to solve the problem. Measured imperfections are introduced into the initial geometry, while the material imperfections are allocated to the nodes of the element cross-section. The GMNIA methodology incorporates nonlinear buckling and post-buckling analyses, which require nonlinear stabilisation techniques to overcome the instability [40,41]. The instability of the post-buckling analysis is consistent with that of the structure. Therefore, nonlinear stabilisations are applied to unstable nodes where large displacements occur. The calculations end when the nonlinear stabilisation cannot recover the problem's stability. Thus, buckling failure coincides with the inability to keep the structure stable. Therefore, the simulation process ends automatically when the column buckles due to instability, making the stopping criterion uniform for all models. The SbcM and CbcM strategies use a 7-step GMNIA, which is explained step by step below.

1. Finite element model. Design the model and determine appropriate boundary conditions.
2. Linear buckling analysis.
3. Initial geometric imperfections.
4. Initial material imperfections. Allocation of internal residual stresses.
5. GMNIA initial phase: mechanical loading. Application of the compressive axial load, flexural moments at the column ends simulating eccentricities, initial temperature, and activation of self-weight stresses.
6. GMNIA second phase: heating. Using the mechanical load from the preceding phase, gradually apply temperature increments of 200 °C/h across the length of the column (L = 2700 mm) until failure occurs.
7. Monitoring displacement results with temperature changes. Record the temperature and displacement at the time of buckling failure.

### 4.1 Finite element model

**Fig. 10:**
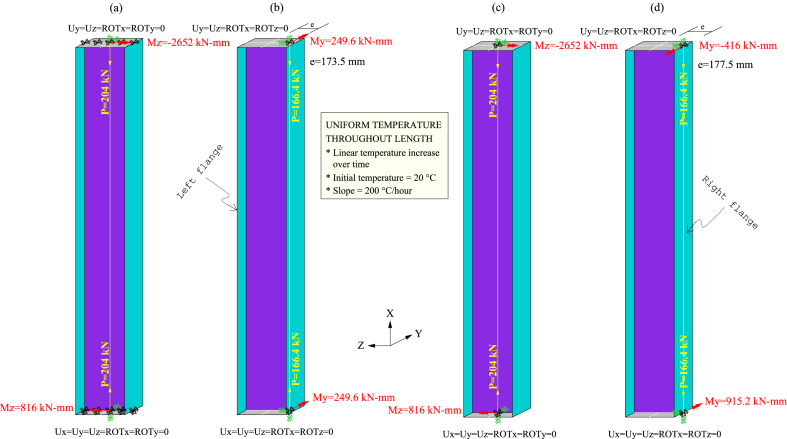
Boundary conditions and loads: (a) SbcM test 3. (b) SbcM test 6. (c) CbcM test 3. (d) CbcM test 6.

Fire tests 3 and 6 on columns with Class 4 steel sections from FIDESC4 were utilised to verify the effectiveness of the proposed modelling strategies. Hence, Single beam-column Models (SbcM) and Cruciform beam-column Models (CbcM) of these two experiments were created to simulate their FTB response. These models were designed to replicate the situations shown in Fig. 10, illustrating the boundary and load conditions. The confinement (which, in FIDESC4 columns, is due to the interaction with the testing machine) is a crucial factor in the temperature-induced stresses affecting the FTB response of the columns. In addition, a good definition of boundary conditions is also essential because they affect the stability of the numerical models. With these criteria in mind, two discretisation strategies for the SbcM models are proposed. For example, in test 3, the compressive load eccentricities act in the weak direction (*y*-axis), so buckling occurs in the *y*-plane (mainly in the web plate). It means that the bending moments due to the eccentricities and the radius of gyration are about the *z*-axis. Hence, the dimension of the cross-section about the *z*-axis, along which the axial compressive load is transmitted to the end of the tested column, should be the largest one, i.e., 450 mm (see Fig. 1, Fig. 2b). This extensive dimension causes the column overlength to be discretised in the *z*-axis and not in the axial direction, as is usually the case. Thus, the SbcM strategy of test 3 is a line model with an I-shaped cross-section with T-ends where the ends are rectangular in cross-section (see Fig. 8a). The compressive axial load is applied at the top end of the column. Also, equivalent moments of Mz = −2652 kNmm at the top end and Mz = 816 kNmm at the bottom end resulting from the measured eccentricities are applied (see Fig. 10, Fig. 13a, b).

At the extremities of the I-shaped column, rotational restraints in the *y*-direction and torsional restraints about the *x*-axis are imposed. That is due to restrictions on the device connecting the specimen to the testing machine. At the lower T-shaped end, all displacements are restrained. Only transverse displacements are restrained at the upper T-shaped end to allow axial displacements, as shown in Fig. 8, Fig. 10a. A link FE representing the interaction with the testing machine is added at the top end of the column. All displacements at the upper end of the link FE are restrained, as shown in Fig. 8, Fig. 13a, b. The compression link FE simulates the testing machine's confinement on the column and functions when thermal expansion activates it.

In SbcM of test 6, the compressive load eccentricities act in the strong direction (*z*-axis), so the buckling occurs in the *z*-*z* plane (mainly in the loaded flange). It means that the flexural moments due to the eccentricities and the radius of gyration are about the *y*-axis. Therefore, the dimension of the cross-section about the *y*-axis, along which the axial compressive load is transmitted to the end of the tested column, should be the smallest one, i.e., 150 mm (see Fig. 3, Fig. 4b). Since this dimension is small, changing the discretisation direction at the column overlength (rectangular in cross-section) in SbcM of test 6 is unnecessary. Therefore, the discretisation direction is continuous along the *x*-axis direction. The compressive axial load of 166.4 kN is applied at the top end, along with two equivalent moments of My = 249.6 kNmm at each end (see Fig. 10, Fig. 13c, d). The load axis is located at an equivalent eccentricity of 173.5 mm, which is the average of two measured eccentricities. Rotational restraints in the *z*-direction and torsional restraints about the eccentric *x*-axis are imposed at the column ends (see Fig. 9, Fig. 13c, d). All displacements are restrained at the bottom end of the column. At the top end, only transverse displacements are restrained. A link FE is added at the top end of the column. All displacements at the link's upper end are restrained, as shown in Fig. 9, Fig. 13c, d.

In CbcM, the discretisation is a grid of rectangular beam FE. The load axis (centric for test 3 and eccentric for test 6) matches the link element axis. The boundary conditions are applied at the ends, on the load axis, as shown in Fig. 8, Fig. 9, Fig. 18. In test 3, rotations about the *z*-axis are released to allow web buckling along the weak *y*-axis. In test 6, *y*-rotations are released to enable web buckling along the strong *z*-axis. In addition, the eccentricities between the axis of the pinned supports and the column axis are included. Hence, in test 3, the compressive axial load of 204 kN is applied on the centric axis along with two equivalent moments of Mz = −2652 kNmm at the top end and Mz = 816 kNmm at the bottom end (see Fig. 10, Fig. 18a, b). In test 6, the load axis is on the centroid of the loaded right flange, which means it is at an equivalent eccentricity of 177.5 (see Fig. 10d). Therefore, a compressive axial load of 166.4 kN on the eccentric axis, along with equivalent moments of My = −416 kNmm at the top end and My = 915.2 kNmm at the bottom end), are applied (see Fig. 10d).

According to the fire tests, the uniform thermal load in the whole column is specified at a controlled rate of 200 °C/h until the buckling failure occurs.

The models utilised the stress-strain ratio of steel at elevated temperatures and incorporated the reduction factors specified in Eurocode 3 [7]. Table 1 provides the yield strength and elastic modulus values of the materials used for modelling the section and overlength at ambient temperature. It was assumed that the Poisson's ratio remains constant at 0.3. The multilinear isotropic temperature-dependent hardening model was incorporated to represent the stress-strain relationships at different temperatures [42]. This material model implicitly accounts for the effect of creep. Temperature-dependent thermal expansion of steel was determined according to Eurocode 3 [7]. Geometric non-linearity is considered to incorporate the effects of large deflections and deformations. All the necessary data for creating the numerical models was extracted from the FIDESC4 research [10].

### 4.2 Linear buckling analysis

A linear elastic buckling analysis is performed to define the initial geometry with imperfections of the numerical models. From this analysis, two buckling modes are identified, consistent with the global and local imperfection profiles measured in the laboratory (refer to Fig. 5). For instance, the global buckling mode with a single wave corresponds to the global imperfection profile of the column. The simulated amplitudes are also retrieved and adjusted using the selected global and local buckling modes to match the measured amplitudes. Thus, for example, the maximum lateral deflection in the *y*-direction of the selected global mode represents the simulated global amplitude. Similarly, the simulated local amplitude for the web is obtained when this maximum deflection is taken from the adopted local mode. The simulated amplitudes are taken from the whole model, excluding the overlength.

### 4.3 Initial geometric imperfections

The displacements of the chosen global and local buckling modes, computed in the prior step, are adjusted and weighted by two factors. These resulting displacement values are then incorporated into the imperfection-free geometry to simulate the imperfect geometry. The first is the scaling factor, which adapts the displacements of the defined buckling modes according to the values of the measured amplitudes. The scaling factor is the quotient of the measured and simulated amplitude. Two factors affect the *y*-displacements of global and local buckling modes of the web, while two more factors affect the *z*-displacements of local modes in the left and right flange. The scaling factors are presented in Equations (2)–(5). The terms G and L in the definition of the factors stand for Global and Local, respectively. In this context, WGSF and WLSF are the global and local scaling factors in the web, while the scaling factors relative to the two flanges are FLSF1 and FLSF2. In these equations, $A_{m}$ represents the measured amplitude while $A_{s}$ is the simulated amplitude. The terms *w*, *lf*, and *rf* refer to the web, left, and right flange. Subscripts *y* and *z* denote simulated imperfections oriented in corresponding directions.
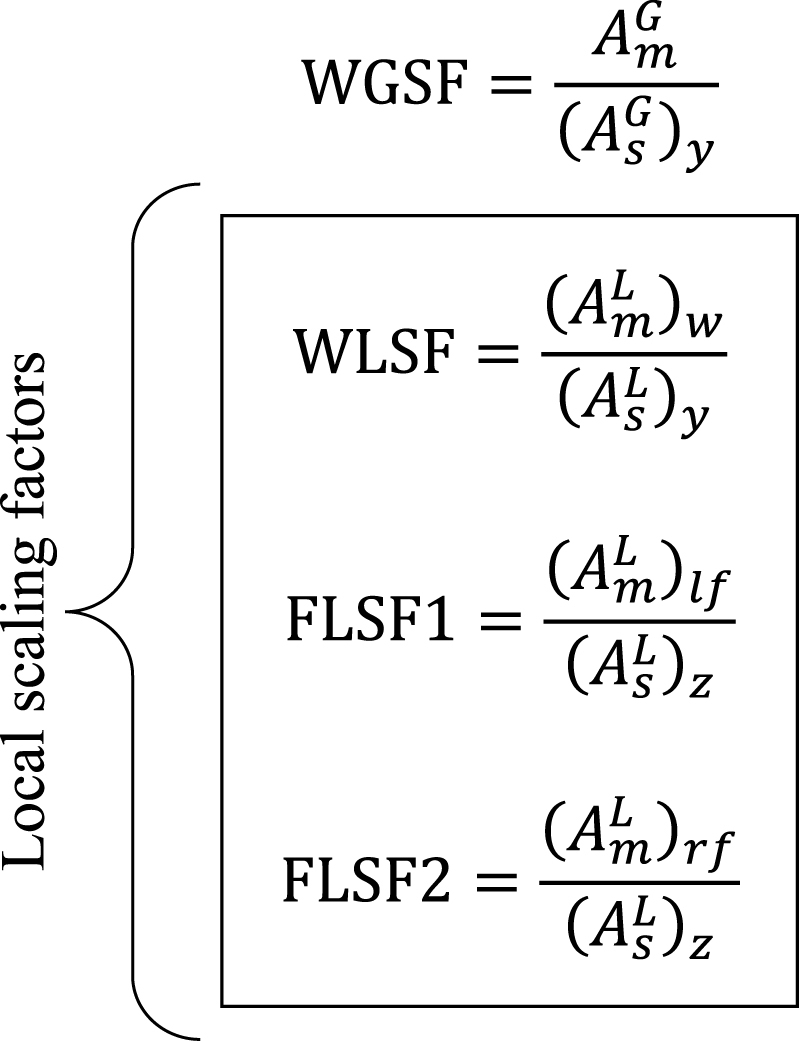


The second is the participation factor, which determines the contribution of the displacements of each selected buckling mode in the generation of the imperfect geometry. In this context, GPF represents the displacement participation factor of the global mode. At the same time, WLPF, FLPF1, and FLPF2 are the displacement participation factors of the local modes. In the proposed numerical modelling strategies, the global and local web participation factors are assumed in halves due to their simultaneous presence in the same direction (see Equation (6)). Meanwhile, the value of the participation factor in each flange is 1, as shown in Equation (7).

(6) $$\text{GPF}=\text{WLPF}=\frac{1}{2}$$

(7) FLPF1=FLPF2=1

As a result, imperfections introduced by the global mode (GI) and the local mode (LI) arise from scaling and weighting the nodal displacements of the global mode (GMS) and those of the local mode (LMS), as detailed in Equations (8), (9). Except for the overlength, global and local imperfections are added to the whole column model.(8)
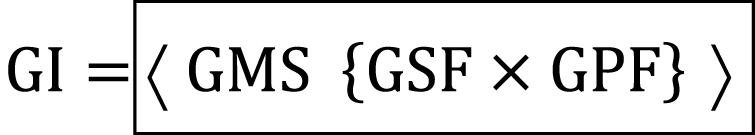
(9)
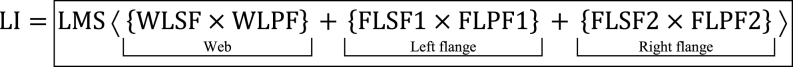


The global imperfections are initially incorporated into the nodal coordinates of the model, excluding the overlength, to generate the initial imperfect geometry (IIG). Then, the same process is carried out for the local imperfections, as illustrated in Equation (10). In this expression, the full geometry coordinates with imperfections included (FGC) are stored in the updated nodal coordinate matrix of the model. The displacements added as imperfections to the geometry are more prominent in the *y*-direction for the web and the *z*-direction for the flanges.(10)
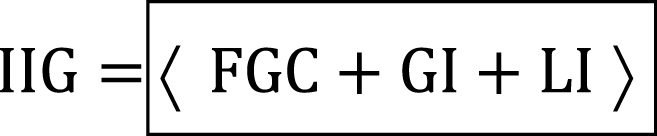


However, due to its nature, the SbcM model requires a single local scaling factor that incorporates the amplitudes of the local imperfections on both the web and flanges, as proposed in Equation (11). In this expression, ${\left(A_{m}^{L}\right)}_{w}$ represents the amplitude of the measured local imperfection of the web while ${\left(\bar{A_{m}^{L}}\right)}_{f}$ indicates the average amplitude of the measured local imperfections of both flanges.

(11) $$\text{LSF}=\frac{{\left[{{\left(A_{m}^{L}\right)}_{w}}^{2}+{{\left(\bar{A_{m}^{L}}\right)}_{f}}^{2}\right]}^{0.5}}{{\left(A_{s}^{L}\right)}_{y}}$$

Therefore, the global and local participation factors for SbcM are taken as 1/2, as shown in Equation (12).

(12) $$\text{GPF}=\text{LPF}=\frac{1}{2}$$

Finally, the local imperfection array (LI*) for SbcM is simplified in Equation (13).(13)
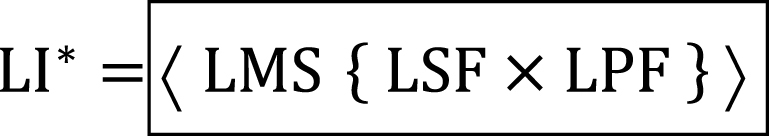


### 4.4 Initial material imperfections

**Fig. 11:**
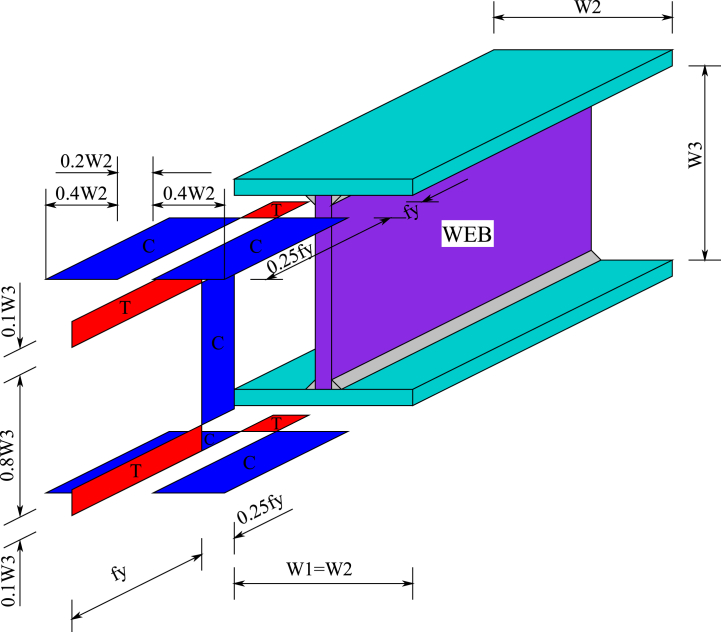
Residual stress distribution of a welded I-profile considered by FIDESC4 [10,43].

Residual stresses exist as an initial stress state at room temperature. Their influence on steel Class 4 profiles exposed to fire is relevant [13]; therefore, they are included in the models before applying mechanical and thermal loads. Axial tensile and compressive residual stresses are introduced into the cross-section of the beam FE [10] starting from the residual stress distribution of a welded I-profile [43], as shown in Fig. 11. The residual stresses are defined based on the yield stresses at ambient temperature listed in Table 1. The red areas in Fig. 11 are tensile stress (T), and the blue regions are compressive stress (C).

### 4.5 GMNIA initial phase: mechanical loading

The initial load is imposed in this first phase. A room temperature (20 °C) is assigned over the entire column length (L = 2700 m) in 1E-08 min. Then, the self-weight is activated, and the axial compressive load and moments by eccentricities are applied, as shown in Fig. 10. The first GMNIA with geometric and material imperfections is run in this phase. The following GMNIA is based on the results of this analysis.

### 4.6 GMNIA second phase: heating

The axial compressive load, moments due to eccentricity, and self-weight remain applied in this second loading phase. In addition, progressive heating over the entire column length from 20 °C at a 200 °C/h rate is specified. In this heating phase, the occurrence of numerical instabilities determines the time and failure temperature of the simulations.

### 4.7 Monitoring displacement results

**Fig. 12:**
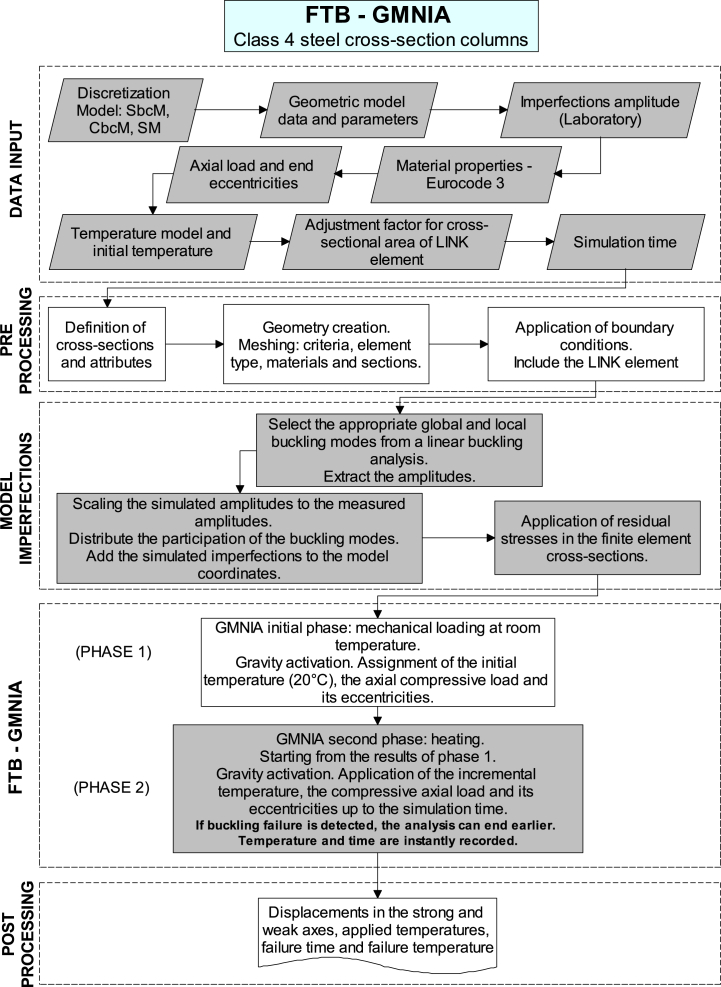
FTB-GMNIA for the FIDESC4 columns.

The time history of the lateral displacements at column mid-height (on the weak and strong axes) and the time history of the total axial displacements are obtained upon completion of the GMNIA of this phase. In test 3, *y*-displacements on the weak axis are monitored at the midpoint of the web. In addition, *z*-displacements on the strong axis at the centre of the left flange are also observed (see Fig. 2, Fig. 6d). In test 6, *z*-displacements are monitored at the centre of the left flange (no loaded), as shown in Fig. 4c. The temperature history over time and the failure time are also reviewed. Finally, the proposed analysis methodology is presented in Fig. 12.

## 5 Implementation of the methodology within the proposed strategies

The FIDESC4 fire tests 3 and 6 were chosen to implement the FTB-GMNIA methodology and validate the proposed SbcM and CbcM modelling strategies.

### 5.1 Single beam-column model (SbcM)

**Fig. 13:**
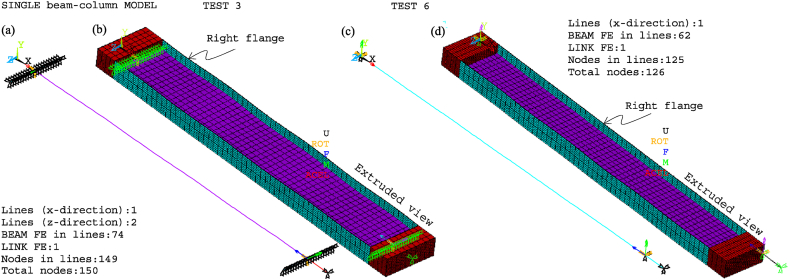
SbcM discretisation and boundary conditions: (a) (b) Test 3. (c) (d) Test 6.

**Fig. 14:**
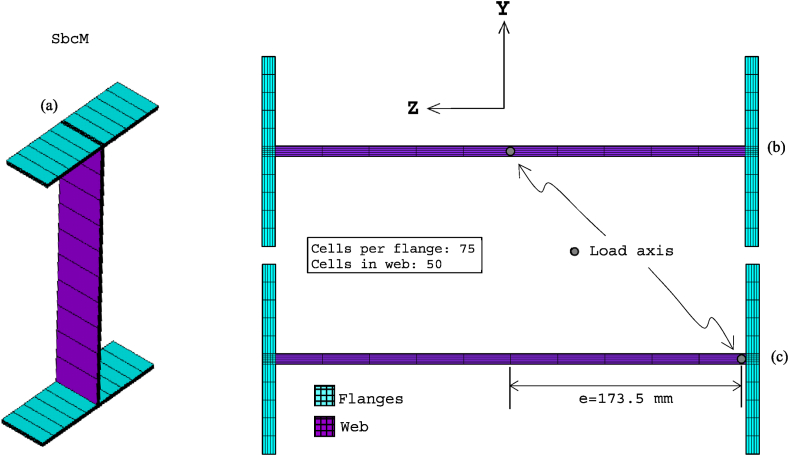
Materials and cells of the I-cross-section: (a) Single 3D beam FE. (b) Test 3. (c) Test 6.

Fig. 13 presents the mesh, loads, moments, and boundary conditions corresponding to tests 3 and 6 for SbcM, while Fig. 14 shows the I-cross-section materials. The SbcM of test 3 comprises a line of 54 beam FE discretising the column and two lines, each of 10 elements, discretising overlength, as shown in Fig. 13a. The SbcM of test 6 is a line of 62 beam FE discretising the column, including that overlength, as shown in Fig. 13c. The discretisation and boundary conditions for both models are shown in Fig. 13b and d. Each beam FE is associated with a cross-section and material relevant to its position, e.g., web, left flange, right flange, or overlength, as shown in Fig. 14a. The column models feature two types of cross-sections and three different materials. That means the welded I-profile cross-section with its web and flange materials and the overlength cross-section with its respective material. Materials and residual stresses are appropriately assigned to the element cross-section cells according to the corresponding cross-section part and residual stress value. In both tests, seventy-five cells per flange and fifty cells in the web were defined, as shown in Fig. 14b and c. The coefficient applied in Equation (1) to adjust the cross-sectional area of the link FE is 0.065 for test 3 and 0.005 for test 6.

The boundary conditions are applied at the ends of the column at the load axis, which is eccentric in test 6. The compression link FE makes it possible to simulate the interaction with the testing machine as the temperature increases. The extruded and non-extruded mesh and the boundary conditions for tests 3 and 6 are shown in Fig. 13. Extruded views in Figs. 13b and d allow distinguishing all model components.

**Fig. 15:**
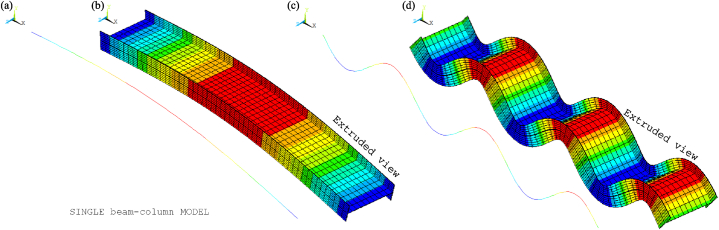
SbcM buckling modes for test 3: (a) (b) Global. (c) (d) Local.

**Fig. 16:**
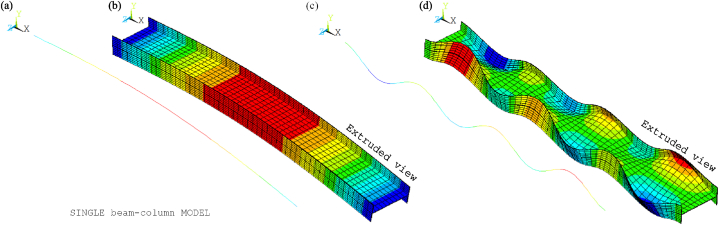
SbcM buckling modes for test 6: (a) (b) Global. (c) (d) Local.

Fig. 15, Fig. 16 illustrate the global and local buckling modes identified from the linear buckling analysis in tests 3 and 6. Fig. 15, Fig. 16a, c show the non-extruded views. Fig. 15, Fig. 16b are the extruded views of the global mode. This buckling mode is distinguished by a single lateral curvature on the weak axis that matches the *y*-direction. Meanwhile, Fig. 15, Fig. 16d are the extruded views of the local modes, where the lateral waves in the *y*-direction are mainly present in the web.

**Fig. 17:**
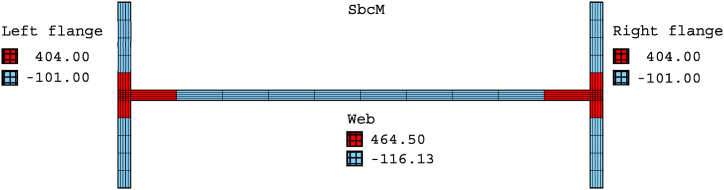
Axial residual stresses for SbcM [MPa].

Equations (2) and (11) determine the two scaling factors, while Equations (8), (13) calculate the imperfections. Finally, Equation (10) adds the calculated imperfections to the model coordinates and obtains the deformed geometry. The initial axial compressive and tensile residual stresses, coloured in red and blue in Fig. 17, are allocated to the cross-section cells.

In the initial phase of GMNIA, all beam FE of the model are subjected to a uniform ambient temperature. In test 3, the measured eccentricities produce equivalent bending moments of Mz = −2652 kNmm at the upper end and Mz = 816 kNmm at the lower end, which are applied to the SbcM model. Also, a compressive axial load of 204 kN is applied at the top-end node of the model (see Fig. 10, Fig. 13a, b). For SbcM of test 6, the equivalent eccentricity (e = 173.5 mm) is the average of two measured eccentricities. Therefore, a compressive axial load of 166.4 kN is applied on the eccentric axis along with equivalent moments of My = 249.6 kNmm at each end (see Fig. 10, Fig. 13c, d).

In the second phase of GMNIA, an incremental uniform temperature (3.33 °C/min) is applied to the cross-section of elements over the length (L = 2700 mm). After the analysis, the following time histories are recorded: transversal deflections in the weak axis (Uy) at the central node of the web, transversal deflections in the strong axis (Uz) at the midpoint of the left flange, and axial displacements (Ux) at the uppermost node of the column.

### 5.2 Cruciform beam-column model (CbcM)

**Fig. 18:**
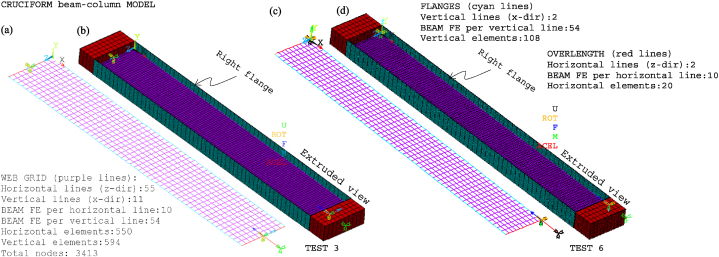
CbcM discretisation and boundary conditions: (a) (b) Test 3. (c) (d) Test 6.

**Fig. 19:**
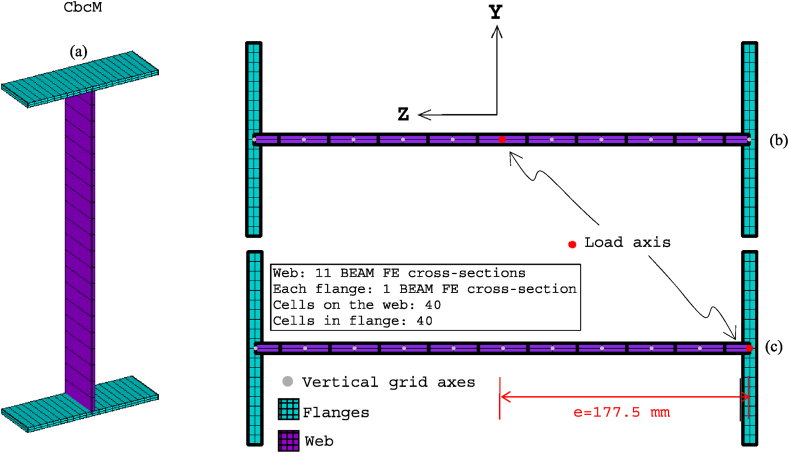
Materials and cells of the I-cross-section: (a) CbcM cross-section discretisation. (b) Test 3. (c) Test 6.

Fig. 18 displays the mesh along with all the model components, loads, moments, and boundary conditions corresponding to tests 3 and 6 for CbcM, while Fig. 19 shows the I-cross-section materials. The CbcM discretisation is a grille of connected beam FE with rectangular cross-sections representing the web (purple coloured in Fig. 18a–d). Additional grid lines idealising the left and right flange (cyan coloured in Fig. 18a and c) are on both sides of the web. The overlength is also represented by grid lines above and below the web (red-coloured in Fig. 18a and c). The beam FE within each model part are assigned the corresponding material and cross-section (refer to Fig. 1, Fig. 3c and Table 1). In test 3, boundary conditions are assigned at both ends of the model, extending along the centroidal axis of the section (see Fig. 18a and b). While in test 6, they are applied on the centroidal axis of the right flange (e = 177.5 mm). Compressive axial loads at the top end and equivalent moments to simulate the load eccentricities are shown in Fig. 10, Fig. 18. A compression link FE simulates the interaction with the testing machine caused by the thermal expansion of the column. The factor used in Equation (1) to calibrate the cross-section area of the link FE is 0.062 for test 3 and 0.130 for test 6.

The welded I-section comprises the rectangular cross-sections of the vertical web grid elements and the flanges, as shown in Fig. 19a–c. The number of cells is determined to appropriately assign the initial residual stresses. As indicated in test 6, the load axis is situated along the right flange (see Fig. 19c).

**Fig. 20:**
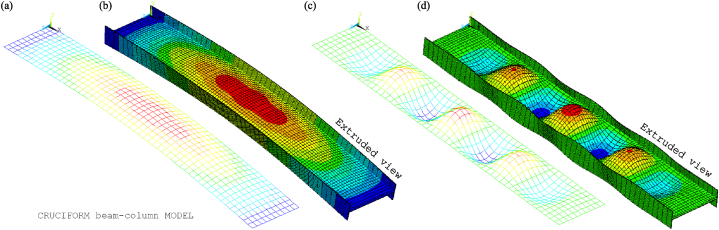
CbcM buckling modes for test 3: (a) (b) Global. (c) (d) Local.

**Fig. 21:**
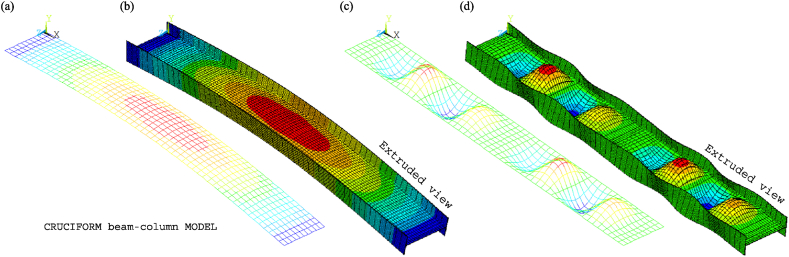
CbcM buckling modes for test 6: (a) (b) Global. (c) (d) Local.

The non-extruded and extruded views of the chosen global and local buckling modes for tests 3 and 6 are shown in Fig. 20, Fig. 21. The global modes are presented in Fig. 20, Fig. 21a, b. They are characterised by a lateral curvature in the weak axis direction. The lateral waves, which represent the local web imperfections, occur in the weak axis direction and are characteristic of the local buckling modes shown in Fig. 20, Fig. 21c, d. Similarly, the waves that appear in the strong axis direction indicate the presence of local flange imperfections. Consequently, the web's maximum lateral deflection in the *y*-direction and the local flange's maximum deflection in the *z*-direction correspond to their respective simulated amplitudes of the global and local buckling modes.

**Fig. 22:**
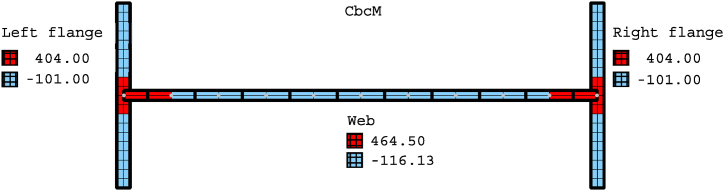
Axial residual stresses for CbcM [MPa].

Equations (2)–(5) determine the scaling and contribution factors, while Equations (8), (9) compute the imperfections. Finally, Equation (10) adds the estimated imperfections to the model coordinates and obtains the deformed geometry. Fig. 22 displays the I-profile, which comprises the element cross-sections of the web and the two flanges. The initial axial compressive and tensile residual stresses indicated in blue and red, respectively, are assigned to the cells of the corresponding element cross-sections.

During the first phase of GMNIA, all beam FE of the model are subjected to a uniform ambient temperature. In test 3, a compressive load of 204 kN is applied along the central axis, with additional equivalent moments of Mz = −2652 kNmm at the top and Mz 816 kNmm at the bottom (refer to Fig. 10, Fig. 18a, b). In test 6, the load axis is located on the centroid of the right flange, resulting in an equivalent eccentricity of e = 177.5 mm. Consequently, a compressive axial load of 166.4 kN is applied at the top end on the eccentric axis. Moreover, equivalent moments of My = −416 kNmm at the top end and My = 915.2 kNmm at the bottom are applied (refer to Fig. 10, Fig. 18c, d).

An incremental, uniform temperature of 3.33 °C/min is applied to the element cross-section along the column until buckling failure occurs. After the analysis, the following time histories are monitored: *y*-displacements in the weak axis at the node at the centre of the web grid, *z*-displacements in the strong axis at the middle of the grid line representing the left flange, and *x*-displacements at the top end node on the load axis (see Fig. 18).

**Fig. 23:**
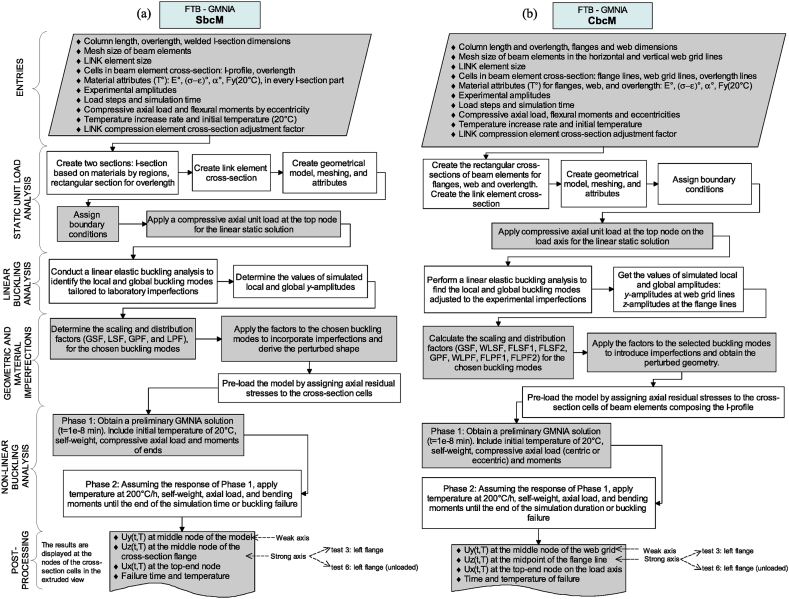
FTB-GMNIA flowchart: (a) SbcM. (b) CbcM.

Fig. 23 depicts a synthesis of how the FTB-GMNIA methodology is implemented in each strategy. The methodology flowchart for SbcM and CbcM is shown in Fig. 23a and b, respectively. These descriptions are tailored to constant cross-section columns.

### 5.3 Numerical validation with shell-based models

**Fig. 24:**
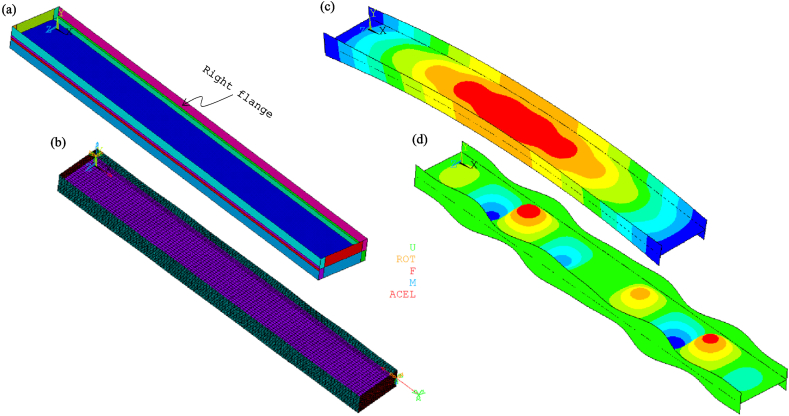
SM for test 6: (a) Geometric model. (b) Meshing and boundary conditions (c) (d) Global and local modes.

Shell-based models for tests 3 and 6 are hereafter referred to as SM. Its construction aims to evaluate the performance of the proposed modelling strategies, SbcM and CbcM, by benchmarking the modelling complexity based on the number of nodes, elements, DOF, and computational times. A linear four-node Reissner-Mindlin shell FE with six DOF/node was used to analyse large rotations and strains nonlinearly. A concise description of implementing the FTB-GMNIA methodology using the SM for test 6 is provided, as it is identical for both tests. Fig. 24 depicts both the geometric and shell models alongside the local and global buckling modes employed to generate imperfections for test 6.

The geometric model for constructing the SM consists of 23 areas corresponding to the residual stress locations, as shown in Fig. 24a. SM discretisation has 7872 shell FE. In addition, *y*-rotations are released on both ends to enable web buckling along the strong *z*-axis. The factor used in Equation (1) to calibrate the cross-section area of the compressive link FE is 0.070 (this factor in test 3 is 0.036). CbcM boundary conditions are similar to the SM because shell FE are on the middle plane of the web and flanges in the model. Boundary conditions imposed on SM are shown in Fig. 24b.

Figs. 24c and d show the global and local buckling modes taken from the linear buckling analysis. Due to its inherent characteristics, the SM can readily scale and merge the two buckling modes following Equations (2) to (9). Moreover, the imperfect initial geometry is created according to Equation (10). The first and second GMNIA phases are the same as those described for the CbcM.

## 6 Validation of the SbcM and CbcM strategies

The two strategies for numerical modelling constant Class 4 cross-section steel columns affected by high temperatures are founded on the GMNIA methodology, which analyses buckling-to-collapse. These strategies are validated by comparing them against the experimental and numerical results of FIDESC4 fire tests 3 and 6, which were subjected to combined axial compression and bending with minor and significant compressive load eccentricities [10]. In addition, the proposed modelling strategies for test 3 are compared against numerical simulations with Bernoulli beam FE using the Eurocode 3 elevated temperature steel constitutive law published in Ref. [25] and an effective stress-based law reported in Ref. [30].

### 6.1 Test 3 validation

**Fig. 25:**
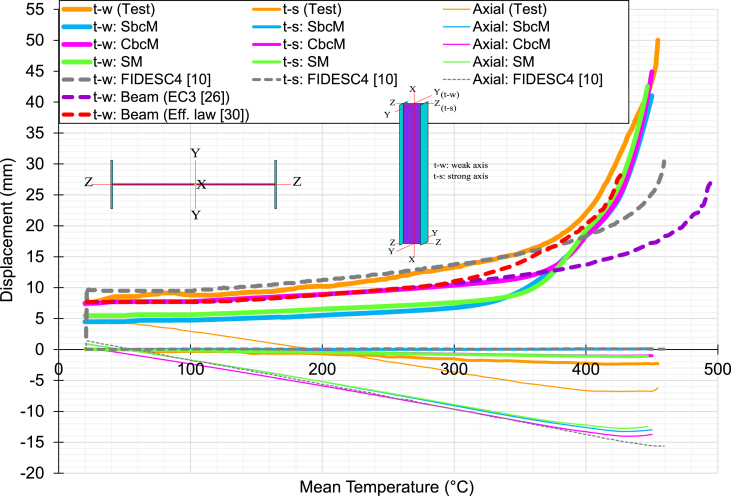
Displacements versus temperature for test 3.

Fig. 25 displays the transverse displacement evolution at the midpoint of the column in the weak axis (t-w) and strong axis (t-s) and the change in the total axial displacement of the column with the steel temperature for test 3. The transverse displacement on the weak axis (*y*-direction) is controlled in the web, and on the strong axis (*z*-direction) in the centre of the left flange. The results of simulations using the SbcM, CbcM, and SM strategies are compared to those of FIDESC4 with shell FE and those published in Refs. [25,30] with Bernoulli beam FE, which incorporate the Eurocode 3 elevated temperature steel constitutive law [25] and an effective stress-based law [30].

Displacement and temperature predictions with the proposed strategies plotted to failure are very close to the test. Furthermore, the predictions of the axial and (t-s) strong axis displacements with the temperature of the proposed strategies do not show significant variations regarding the simulations used to validate. Therefore, the comparisons focus on the column's midpoint (t-w) displacements along the weak axis. Thus, it is observed that the CbcM is the one that most closely matches the test-to-failure behaviour. Furthermore, SbcM and SM also very well predict the displacement behaviour in the near-failure zone. However, the best option for predicting failure temperature and displacement is SbcM because it is a simple model with few finite elements and low computational cost that gives satisfactory results.

The ratio of the simulated failure temperature to the test 3 failure temperature is calculated in Table 4. The results indicate that SbcM and CbcM are accurate within a 0.44% error, while SM predicts lower failure temperatures and FIDESC4 simulation [10] overestimates them. Therefore, SbcM is the best option to predict test 3's failure temperature due to its low cost and simplicity.

**Table 4:** Comparison of simulated and test 3 failure temperatures.

	Time of failure (min)	Temperature of failure *T*_*f*_ (°C)	$\text{Ratio}=\frac{T_{f}^{\text{Num}}}{T_{f}^{\mathrm{Exp}}}$
Test 3	129.6	452.0	–
SbcM	129.0	450.0^a^	0.996
CbcM	129.0	450.0^a^	0.996
SM	128.0	446.7	0.988
FIDESC4 [10]	131.7	459.0	1.015

a Reference temperature.

A reference temperature value is established for comparing results as SbcM, CbcM, and SM predict lower failure temperatures. This reference temperature is the SbcM (or CbcM) failure temperature since it is closest to the test. Table 5 displays the measured and simulated (t-w) weak axis displacements around test 3 failure temperature and the percentage change between these two displacements in absolute value. Deflections in Table 5 are recorded at the reference temperature if the simulated failure temperature exceeds the test temperature; otherwise, they are recorded at the simulated failure temperature.

**Table 5:** Variation of simulated deflection along the weak axis for test 3.

	Temperature [°C]	(t-w)_test_ [mm]	(t-w)_simulated_ [mm]	|Variation| [%]
SbcM	450.0^a^	43.483	41.015	5.7
CbcM	450.0^a^	43.483	44.911	3.3
SM	446.7	41.377	42.553	2.8
FIDESC4 [10]	450.0^a^	43.483	25.200	42.0
Beam (EC3 [25])	450.0^a^	43.483	17.234	60.4
Beam (Eff. Law [30])	426.4	31.871	28.224	11.4

a Reference temperature.

Comparisons include displacements obtained from simulations based on Bernoulli beam FE with Eurocode 3 [25] and the effective law proposed in Ref. [30]. As a result, *y*-displacement predictions close to the failure temperature are reasonably good in the SbcM, CbcM, and SM strategies. However, the SbcM is again confirmed as the best option due to its simplicity and low computational cost.

**Fig. 26:**
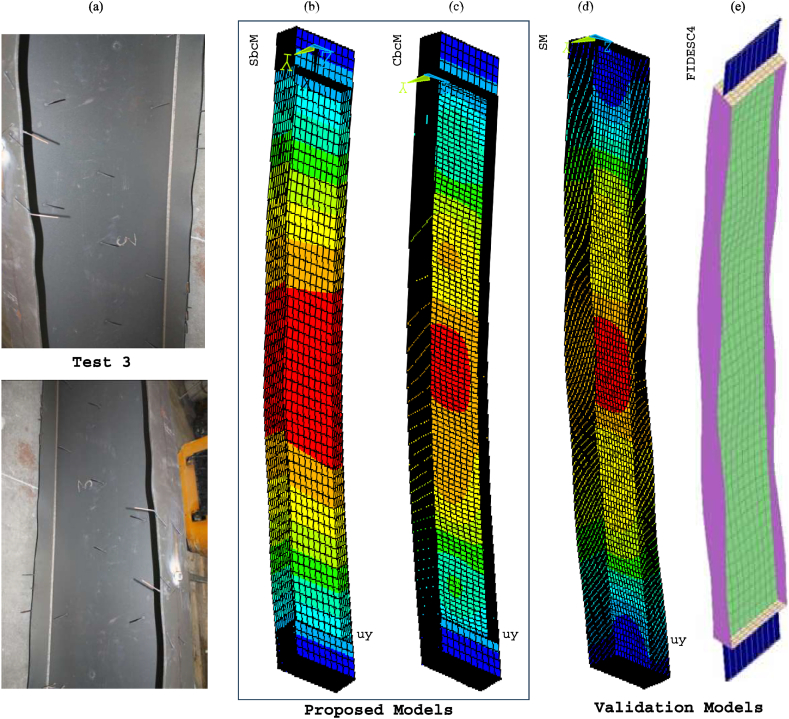
Failure modes: (a) Test 3. (b) SbcM. (c) CbcM. (d) SM. (e) FIDESC4 [10].

**Fig. 27:**
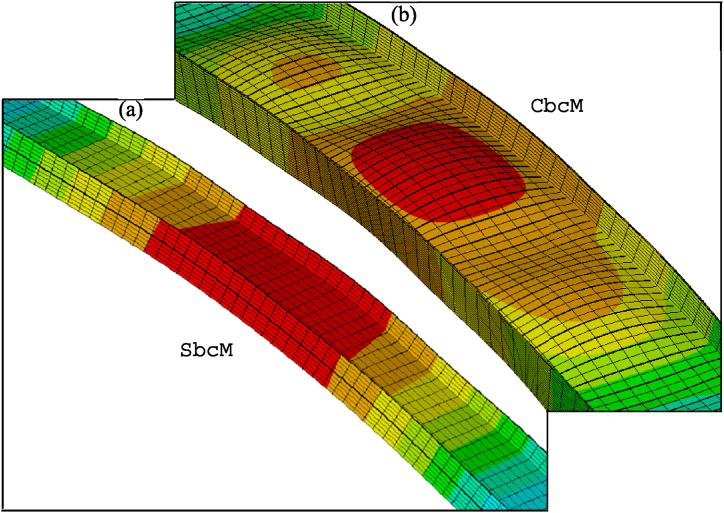
Details of failure mode for test 3: (a) SbcM. (b) CbcM.

Fig. 26 compares the failure modes of test 3 and SbcM, CbcM and SM modelling strategies. In Fig. 26e, the failure mode of the FIDESC4 shell simulation is presented as a reference. Fig. 27 presents detailed views of the failure modes for SbcM and CbcM. The flanges on the failed column in Fig. 26a exhibit minor buckling, as does the length of the column. The SM failure mode in Fig. 26d simulates local buckling in the flanges. The SbcM and CbcM failure modes in Figs. 26b and c reproduce the global failure as well as slight local failures (see details in Fig. 27a and b). These strategies are advantageous over SM due to their easiness and fewer elements required for discretisation, resulting in lower computing costs. Fig. 27a shows how SbcM can replicate local buckling and flanges in the web despite being a single-line model. Therefore, simulating FTB in Class 4 steel columns with small compressive load eccentricities under fire can be done simply, accurately, and computationally cheaply using SbcM.

### 6.2 Test 6 validation

**Fig. 28:**
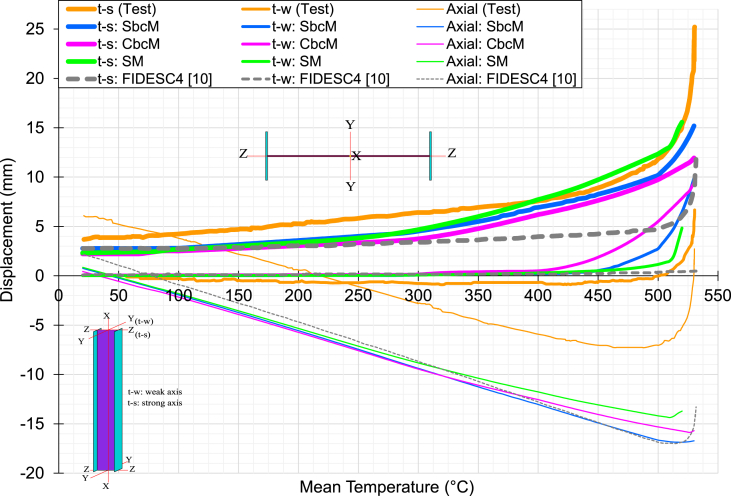
Displacements versus temperature for test 6.

In Fig. 28, the deflection results for test 6 are shown. The displacements of the column midpoint on the strong axis (t-s) are significant in the buckling failure due to the high compressive load eccentricity that introduces significant bending stresses in that direction. For this reason, the comparisons are made on that axis (displacements on the strong axis are the thicker curves). The best deflection predictions on the strong axis are those of the proposed SbcM and CbcM modelling strategies. However, SbcM stands out in the near-failure region as a good predictor of the displacement and the failure temperature. The offset of the axial curves indicating the shortening of the column end is probably because the axial displacement was measured on the distribution beam. Despite this, the simulated axial displacement with steel temperature behaviour is similar to the measured in the test.

The ratio of the simulated failure temperature to the test 6 failure temperature is calculated in Table 6. The results indicate accurate prediction by SbcM and CbcM, underestimation by SM and overestimation by FIDESC4 [10]. Table 7 presents the measured and simulated (t-s) strong axis displacements at or near failure temperature and the percentage change between these two displacements in absolute value. Deflections in Table 7 are recorded at the test failure temperature if the simulated failure temperature exceeds that value; otherwise, they are recorded at the simulated failure temperature. As a result, *z*-displacement predictions near the failure temperature are reasonably good in the SbcM and CbcM if the high asymptotic behaviour of the curves in the failure region is considered. However, the best is the SbcM due to its simplicity and low computational cost.

**Table 6:** Comparison of simulated and test 6 failure temperatures.

	Time of failure (min)	Temperature of failure *T*_*f*_ (°C)	$\text{Ratio}=\frac{T_{f}^{\text{Num}}}{T_{f}^{\mathrm{Exp}}}$
Test 6	129.6	530.0	–
SbcM	129.0	530.0	1.000
CbcM	129.0	530.0	1.000
SM	128.0	520.0	0.981
FIDESC4 [10]	131.7	531.5	1.003

**Table 7:** Variation of simulated deflection along the strong axis for test 6.

	Temperature [°C]	(t-s)_test_ [mm]	(t-s)_simulated_ [mm]	|Variation| [%]
SbcM	530.0	22.638	15.189	32.9
CbcM	530.0	22.638	11.937	47.3
SM	520.0	15.135	15.561	2.8
FIDESC4 [10]	530.0^a^	22.638	8.825	61.0

a Test 6 failure temperature.

**Fig. 29:**
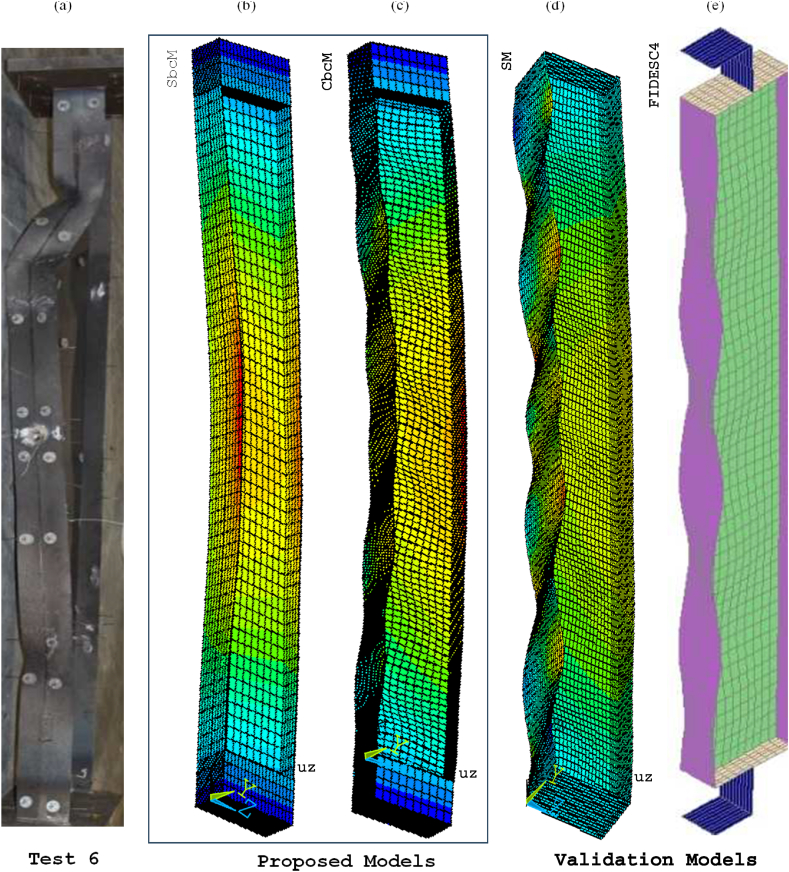
Failure modes: (a) Test 6. (b) SbcM. (c) CbcM. (d) SM. (e) FIDESC4 [10].

**Fig. 30:**
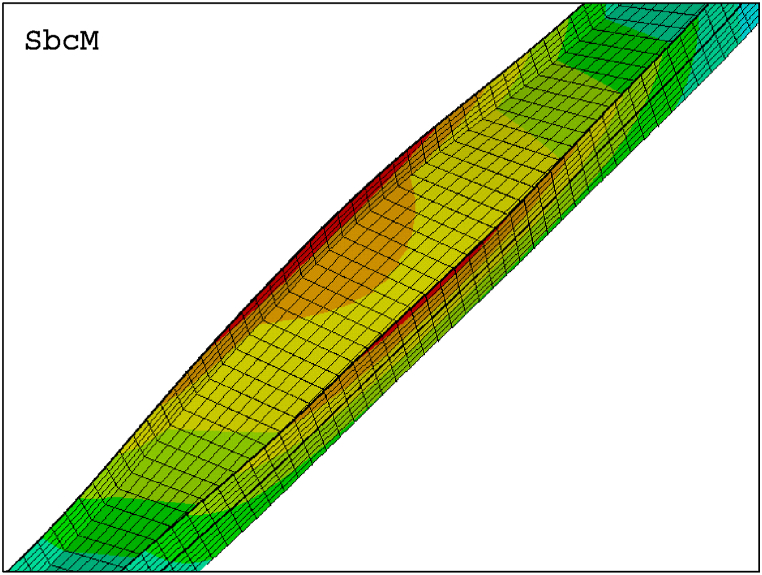
Detail of SbcM failure mode for test 6.

Fig. 29 compares the failure modes of test 6 and SbcM, CbcM and SM modelling strategies. In Fig. 29e, the failure mode of the FIDESC4 shell simulation is shown as a reference. The loaded right flange on the failed column in Fig. 29a shows major buckling failures along the column. The reason is the high compressive load eccentricity. The SbcM failure mode in Fig. 29b replicates the global failure and slight local failures (see details in Fig. 30). Meanwhile, CbcM and SM failure modes in Figs. 29c and d simulate the global failure as well as the local failures on the loaded right flange. However, the advantage of SbcM and CbcM is the reduction in elements, lowering computational cost compared to SM.

**Fig. 31:**
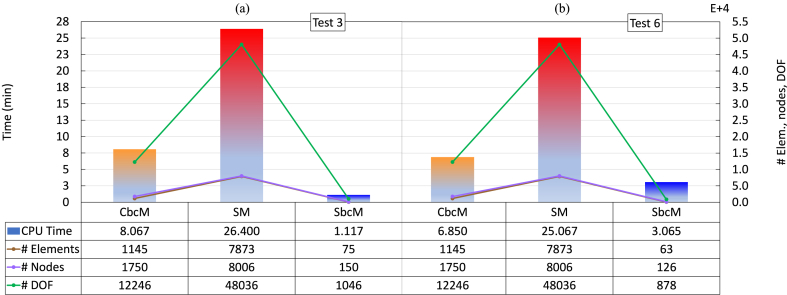
CPU time associated with the number of nodes, elements, and DOF: (a) Test 3. (b) Test 6.

To conclude, the effectiveness of each strategy is demonstrated by the CPU time spent, which is associated with the number of nodes, elements, and DOF used (see Fig. 31). The CPU time in Figs. 31a and b is the total time spent executing all steps of the GMNIA for determining the FTB strength of steel beam-columns with Class 4 cross-sections exposed to fire using each modelling strategy summarised in Fig. 13, Fig. 14.

In tests 3 and 6, the SbcM had the shortest CPU time (see Fig. 31a and b). The reason is the enormous savings in elements, nodes, and DOF. For example, in test 6, using an SbcM model with 63 beam FE gives accurate results for large compressive load eccentricities instead of an SM model with 25,067 shell FE. CbcM effectively detects global and local buckling failures, and it reduced times related to SM by 69.44% in test 3 and 72.67% in test 6. In conclusion, the SbcM proves to be the most efficient method for forecasting the FTB response of Class 4 cross-section steel columns in fire. This achievement is due to its ability to reduce computational costs, accurately calculate failure temperature, and precisely validate displacements for both small and large compressive load eccentricities. The simulations were run on a mobile workstation equipped with a 64-bit Intel Xeon processor operating at 2.71 GHz and 32 GB of RAM. CPU times were obtained using a 6-core physical-MPI distributed-memory parallel computation setup.

## 7 Conclusions, limitations and future work

Two modelling strategies are proposed to forecast the FTB response of fire-affected steel beam-columns with constant Class 4 cross-sections under eccentric compressive axial loads. These use Timoshenko beam FE and are based on GMNIA, including geometrical and material imperfections and induced stresses by thermal expansion. The first modelling strategy (SbcM) idealises the beam-column as a collection of connected beam FE, and the second (CbcM) as a grid of connected beam FE. In addition, the induced thermal stresses are simulated through a link FE in compression. Experimental and numerical simulations of two fire tests on Class 4 cross-section steel columns subjected to combined compression and bending from FIDESC4 investigation [10] satisfactorily validate the two strategies for small and large compressive load eccentricities. Based on the conducted research, the following deductions are drawn:

- SbcM and CbcM accurately simulate the displacement evolution with the temperature as well as the displacement and the failure temperature of the Class 4 steel cross-section beam-columns under fire for both small and sizeable compressive load eccentricities and avoid using high-cost computational models with shell FE. This simplification is a significant advantage over shell-based simulations and other beam fibre-type proposals, yielding only satisfactory results for small compressive load eccentricities.
- SbcM discretises the steel beam-column through a beam FE line representing the load axis, at the ends of which the boundary conditions are applied. The beam-column is subjected to an equivalent loading system consisting of the compressive axial load and a flexural moment at each end, simulating the compressive load eccentricities. In addition, SbcM includes a compressive link FE located at the top end of the model to introduce the induced stresses due to thermal expansion. SbcM correctly predicts the displacement evolution with the steel temperature (in the weak and strong axes), the failure displacements, and the failure temperature for small and large compressive load eccentricities. Its construction is straightforward, and the model's compact size is attributed to its minimal number of nodes, elements, and DOF. Thanks to its modest size, this model ensures swift results processing, leading to efficient computation times. Moreover, it can adeptly simulate slight local buckling failures.
- CbcM employs a grid of interconnected beam FE to discretise the web and uses lines of interconnected beam FE to simulate the flanges to enhance the detailed capture of local buckling failures. It simplifies the construction of the finite element model and effectively simplifies the computational costs associated with traditional shell models used for FTB analyses of fire-affected Class 4 steel cross-section columns. CbcM also satisfactorily predicts the displacement evolution with the temperature (in the weak and strong axes), the failure displacement, and the failure temperature for small and large compressive load eccentricities.
- SbcM and CbcM correctly capture the FTB response with a shorter computational time than shell models. However, the SbcM is better because its simplicity enables faster and more accurate analysis of complex fire engineering problems that require many simulations, such as probabilistic and optimisation studies.
- Satisfactory SbcM and CbcM results demonstrate that the Eurocode 3 elevated temperature structural steel constitutive law and total cross-section area are adequate to simulate the local buckling of thin-walled steel beam-columns under fire correctly. In other words, the steel member's nominal bending and axial stiffness, instead of their reduced values, give correct results for the studied problem. In conclusion, correctly predicting the FTB response of thin-walled steel beam-columns under fire is possible with a good discretisation model using Timoshenko beam FE featuring seven DOF, boundary conditions adapted to the phenomenon's reality, and a type and analysis options appropriate to the nature of the problem (i.e., a highly nonlinear problem with initial conditions).
- The methodologies used for modelling the SbcM and CbcM strategies can be adapted to other software capable of performing the multi-physics modelling and nonlinear analyses that are intrinsic to the instability problems of Class 4 cross-section steel beam-columns subjected to high temperatures. Also, a three-dimensional Timoshenko beam FE is necessary to spatially represent the domain, with a pseudo-mesh in the cross-section to account for material heterogeneity and the application of initial stresses within the section.
- The validity of the proposed modelling strategies is limited to slender steel beam-columns with constant Class 4 cross-sections at elevated temperatures without lateral restraints.

Future work should address developing new modelling strategies for thin-walled steel elements with variable cross-sections under fire conditions and applying these methodologies to more complex fire engineering problems involving the execution of many simulations, such as probabilistic and optimisation designs and parametric studies.

## CRediT authorship contribution statement

**Myriam R. Pallares-Muñoz:** Conceptualization, Formal analysis, Investigation, Methodology, Validation, Visualization, Writing – original draft, Writing – review & editing. **Ignacio Paya-Zaforteza:** Project administration, Supervision, Writing – original draft, Funding acquisition. **Antonio Hospitaler-Pérez:** Project administration, Supervision.

## Declaration of competing interest

The authors declare that they have no known competing financial interests or personal relationships that could have appeared to influence the work reported in this paper.
